# Exploring the Therapeutic Impact of Repetitive Transcranial Magnetic Stimulation (rTMS) in Individuals With Alzheimer's Disease: A Comprehensive Narrative Review

**DOI:** 10.7759/cureus.84885

**Published:** 2025-05-27

**Authors:** Rochaknaveen Bains, Hamna Talha Peracha, Sai Harini Chandrasekaran, Kambam Sneha Reddy, Divyanshi Vijay Kumar, Ananya Prakash, Manjeet Singh, Misbah Kamal Khan, Shree Rath

**Affiliations:** 1 Psychiatry, St. Francis Emory Healthcare, Columbus, USA; 2 Internal Medicine, Fatima Jinnah Medical University, Lahore, PAK; 3 General Medicine, Omandurar Government Medical College, Chennai, IND; 4 Medicine, Bukhara State Medical Institute, Bukhara, UZB; 5 Internal Medicine, Smt. Nathiba Hargovandas Lakhmichand Municipal Medical College, Gujarat, IND; 6 Medicine, Vydehi Institute of Medical Sciences and Research Centre, Bangalore, IND; 7 Internal Medicine, Liaquat National Hospital and Medical College, Karachi, PAK; 8 Medicine, Peoples University of Medical and Health Sciences, Nawabshah, PAK; 9 Internal Medicine, All India Institute of Medical Sciences (AIIMS), Bhubaneswar, IND

**Keywords:** alzheimer's disease, behavioral and psychological symptoms of dementia (bpsd), non-pharmacological interventions, repetitive transcranial magnetic stimulation (rtms), theta burst stimulation (tbs)

## Abstract

Alzheimer’s disease (AD) is a progressive neurodegenerative disorder marked by cognitive deterioration and behavioral symptoms, significantly impacting patients and caregivers. Behavioral and psychological symptoms of dementia (BPSD), including agitation, depression, and apathy, are common in AD and complicate its management. Conventional treatments, such as cholinesterase inhibitors and antipsychotics, offer limited benefits and often carry adverse effects. Repetitive transcranial magnetic stimulation (rTMS), a non-invasive neuromodulatory technique, has emerged as a promising alternative by modulating cortical excitability and promoting neuroplasticity. This narrative review aims to evaluate the current literature on the therapeutic effects of rTMS in AD, with a focus on its impact on cognitive function and behavioral symptoms. A comprehensive literature search was conducted across PubMed, MEDLINE, PsycINFO, Embase, and the Cochrane Library. Studies were selected based on predefined inclusion and exclusion criteria, with quality assessed using the Cochrane Risk of Bias Tool and the Newcastle-Ottawa Scale. Findings indicate that high-frequency rTMS can improve memory, language, and executive functions, while also reducing neuropsychiatric symptoms such as agitation and depression. Mechanistically, rTMS appears to influence cortical plasticity, neurotransmitter regulation, and neurotrophic factor expression. Despite these encouraging outcomes, heterogeneity in study design, stimulation protocols, and patient populations limit generalizability. Further research through large-scale, standardized, multicentric trials is necessary to confirm its efficacy and define optimal therapeutic parameters. In conclusion, rTMS holds promise as an adjunctive intervention in AD, potentially improving cognitive and behavioral outcomes while offering a favorable safety profile.

## Introduction and background

Alzheimer's disease (AD) is a neurodegenerative disorder marked by cognitive and behavioral impairments that significantly interfere with social and occupational functioning [1]. It stands out as the most common form of age-related dementia, presenting a substantial public health challenge by accounting for roughly 70% of cases in people between 60 and 85 years old (WHO, 2021) [2]. These symptoms contribute to diminished quality of life, heightened rates of hospital admissions, and additional strain on caregivers [1,3]. Hence, early diagnosis is a principal goal in dementia care (WHO, 2019) [4].

Learning nouns activates the left fusiform gyrus while learning verbs engages other regions, such as the left inferior frontal gyrus and part of the left posterior medial temporal gyrus. Specifically, new nouns activate the left fusiform gyrus, and new verbs activate the left posterior medial temporal gyrus, which is associated with semantic and conceptual aspects. Processing grammar is handled by the inferior frontal gyrus [5]. Consequently, transcranial magnetic stimulation (TMS) has emerged as a promising possibility for treating AD. The repetitive transcranial magnetic stimulation (rTMS) strategy has been tested most frequently and has shown positive responses, consistently demonstrating a persistent enhancement in cortical excitability following repetitive high-frequency stimulation. This involves long-term potentiation (LTP)-like changes in synaptic strength, widely presumed to be a key cellular mechanism of learning and memory [6].

Management of cognitive and behavioral symptoms in AD often relies on symptomatic treatment, which remains the primary approach in everyday clinical practice. Two categories of drugs are approved for treating AD: cholinesterase inhibitors and partial N-methyl D-aspartate (NMDA) antagonists [7]. Newer techniques like monoclonal antibody therapy have shown promising responses, while genetic protein-targeted therapies focusing on SNAP-25 and APOE4 proteins, which play roles in regulating neural tissue plasticity, have also shown promising outcomes [7,8]. Non-drug interventions, such as physical therapy, are being utilized [9]. rTMS provides a non-invasive and safe therapeutic avenue for neurological and psychiatric disorders, demonstrating the potential to enhance cognitive abilities. rTMS, a mode of TMS, involves applying repeated magnetic signals to the cortex, generating an accumulation of excitatory postsynaptic potentials and regulating cognitive function activity. It is capable of modulating cortical excitability and inducing long-lasting neuroplastic changes. Currently, rTMS is the most commonly used method, capable of altering cortical inhibition and excitation based on the stimulation frequency [9-15]. This review evaluates the current literature on rTMS in AD, focusing on its effects on cognition and behavior.

## Review

Methodology

This Narrative review explores the therapeutic effects of rTMS in individuals with AD. Through a rigorous methodology, encompassing extensive literature searches, stringent inclusion and exclusion criteria, and data extraction, the review identifies and synthesizes relevant studies from PubMed, MEDLINE, PsycINFO, Embase, and Cochrane Library databases for the last 25 years. The literature search included peer-reviewed English language studies assessing rTMS effects on cognition and/or behavioral symptoms in AD. Excluded were non-human studies, reviews, editorials, abstracts, and those lacking relevant outcomes. Keywords included “rTMS,” “repetitive transcranial magnetic stimulation,” “Alzheimer’s disease,” “cognition,” “behavioral symptoms,” and “treatment.” Two independent reviewers screen titles, abstracts, and full-text articles to extract data on study characteristics, rTMS parameters, outcome measures, and key findings [16]. Narrative synthesis techniques are used to summarize findings and explore potential variations and the robustness of the review. Through this comprehensive approach, the review aims to shed light on the therapeutic potential of rTMS as a neuromodulatory intervention in AD management.

Alzheimer's disease

AD stands as a profound neurodegenerative ailment, leading to progressive memory deficits and cognitive impairment, ultimately culminating in death within five to 20 years of symptom onset [17,18]. While various factors like genetics and lifestyle influence one's susceptibility to AD, age emerges as the predominant risk factor, with AD uncommon before age 65 and exponentially rising afterward, affording nearly a 40% likelihood of AD by age 85. Presently, over five million individuals in the United States and potentially exceeding 30 million worldwide grapple with AD, with projections indicating a substantial surge due to demographic shifts favoring an aging population [19,20]. AD manifests initially through subtle challenges in memory, learning, and executive function, labeled as mild cognitive impairment (MCI). Over time, these difficulties intensify, rendering patients increasingly reliant on assistance. Ultimately, caregivers confront overwhelming hurdles in meeting the patient's basic needs, preventing wandering, and managing disruptions in the sleep-wake cycle, often necessitating nursing home care for late-stage AD patients.

Epidemiology

The prevalence and impact of AD are escalating due to the growing elderly population. The Centers for Disease Control projects a substantial rise in individuals over 65 by 2030, potentially reaching nearly a billion [19]. In the United States alone, it is estimated that by 2050, over 13.8 million people will have AD dementia, with a significant portion aged 85 and above [19]. From 1990 to 2019, the AD burden increased worldwide, with high alerts in high-SDI areas, especially among elderly populations [20].

Given that the onset is closely related to age, the disease burden is expected to increase with the aging population and increased life expectancy, escalating demands for financial resources for formal and informal caregiving, nursing home costs, healthcare spending, and caregiver expenses [20]. Medicare payments for services from ages 65 and older with AD or other dementias are almost three times those of payments to beneficiaries without these conditions. Total payments in 2023 for health care services for people aged 65 and older with dementia are estimated to be $345 billion [21]. The annual global cost of dementia is now above US$1.3 trillion and is expected to rise to US$2.8 trillion by 2030 [22]. Hence, implementing preventive strategies to delay AD onset is crucial for reducing the global economic burden and societal strain. Monitoring AD risk factors closely is integral to these mitigating efforts.

Risk Factors Affecting Alzheimer's Disease Onset

Old age remains the primary risk factor for AD, but various other factors contribute to its onset and progression. These factors fall into two main domains: pre-existing conditions or diseases and lifestyle choices. Pre-existing conditions include obesity, vascular risk factors, and insulin resistance among others [23-26]. Lifestyle factors encompass the subdomains of physical activity, diet, mentally draining tasks, and stress [27-33].

Clinical features of Alzheimer's disease

AD is characterized by cognitive and functional decline, along with various non-cognitive or behavioral manifestations. The symptomatic phase is divided into mild, moderate, and severe stages, with a progressive decline in cognition and function. The mild stage (stages 1, 2, and 3) involves a gradual decline in cognitive and functional abilities, with neuropsychiatric symptoms (NPS) beginning to emerge [34]. In the pre-dementia or early-stage dementia (stages 1, 2, 3) phase, a person can still live independently and may not exhibit obvious memory loss or difficulty completing regular tasks. Mild dementia symptoms mimic episodes of age-related forgetfulness [35]. The moderate stage of dementia (stages 4 and 5) is characterized by noticeable gaps in memory and thinking, and individuals typically require assistance. Major personality and behavioral changes, including compulsive and repetitive behaviors, may also occur [36]. Severe dementia (stages 6 and 7) is caused by progressive cognitive and functional impairment, leading to complete dependence on basic activities of daily living (ADLs) [37].

Frequency of Psychiatric and Behavioral Symptoms in Alzheimer's Disease and Non-Alzheimer's Disease Dementias

Psychiatric symptoms are prevalent across various types of dementia, including AD, frontotemporal dementia (FTD), dementia with Lewy bodies (DLB), vascular dementia (VaD), and Parkinson’s disease dementia (PDD). The reported types of these symptoms vary widely due to differences in diagnostic criteria, patient populations, dementia severity, measurement instruments, onset of symptoms, and the nature of symptoms [38]. The Pain Assessment in Advanced Dementia (PAINAD) or Face, Legs, Activity, Cry, Consolability (FLACC) scales and the Alzheimer’s Disease Assessment Scale - Cognitive Subscale (ADAS-Cog) are clinical tools for evaluating the reliability and effectiveness of cognitive changes in AD [38]. The pattern of psychiatric symptoms differs among dementia types, aiding in diagnosis. About 63 studies conducted in 2015 revealed that over 40% of individuals diagnosed with AD will show depression as a symptom, compared to 10-15% of the general population [39]. A 2021 meta-analysis of 48 studies revealed that anxiety plays a role with about a 39% prevalence rate in AD.

It is estimated that up to 50% of AD patients will experience characteristics of psychosis throughout their diagnosis, although the severity and duration of psychosis vary among individuals. Aggressive behaviors occur in approximately 30% of patients with AD throughout their disease, with apathy being the most common symptom, ranging up to a 53-92% prevalence rate [40].

Conversely, affective symptoms like depression and anxiety may be more prevalent in VaD (32% and 18%) compared to AD (20% and 8%). FTD is characterized by early personality changes, with nearly all patients experiencing psychiatric and behavioral symptoms. Disinhibition and apathy distinguish FTD from AD, with severe apathy and disinhibition being key discriminators. DLB and PDD commonly present with psychiatric symptoms, including visual hallucinations (DLB 72; PDD 50%) and delusions. Depression, anxiety, and apathy are also common but less characteristic, occurring in AD and VaD as well. These findings underscore the importance of recognizing psychiatric symptoms in dementia diagnosis and management [41].

Etiology of Psychiatric and Behavioral Symptoms in Dementia and Alzheimer's Disease

Behavioral and psychological symptoms of dementia (BPSD) include changes in perception, thoughts, mood disorders, aberrant motor behavior, agitation, and irritability, which are found in all dementia syndromes, including AD. However, these symptoms can vary between different diagnoses. BPSD tends to be influenced by a complex interplay of psychosocial and biological factors and underlying disease mechanisms [42].

A potential overlap of pathophysiological mechanisms between different dementia syndromes, including AD, is noted. Agitation in AD is associated with damage to the insula, anterior cingulate cortex, frontal cortex, and amygdala. Medial frontal and frontoinsular degeneration is common in FTD, characterized by disinhibition and poor social functioning. Hence, there might be a pathophysiological link or overlap between behavioral abnormalities observed in FTD and the presence of agitation in AD [40]. Psychiatric symptoms like apathy, agitation, depression, and psychosis being core features reveal some links between these symptoms and AD. The general AD pathology includes Aβ and APOE4 as their strongest risk alleles. Studies have revealed that early-onset depression is associated with a higher serum Aβ40/Aβ42 ratio, suggesting their link. Aggression and AD are associated based on the well-established correlation between frontal lobe serotonergic dysfunction, suggesting serotonin transporter polymorphisms (specifically 5-HT2A and 5-HT2C) linkage to this theory [43]. Additionally, autopsy and biopsy studies of the brains of individuals with AD have shown decreased levels of serotonergic receptors [43]. Dopamine receptor allele polymorphisms have shown a complex association with psychotic symptoms [44]. Another study concluded that the risk genes for AD associated with NPS include CD33 and EPHA1 for mood disorders related to AD and SORL1 (sortilin-related receptor 1 gene) for frontal symptoms. Additionally, polygenic risk scores (PRSs) have been linked to depression in late-onset AD [45-47].

The literature suggests a multifactorial etiology for psychiatric and behavioral symptoms in dementia and AD. Additionally, a study comprehended that a low level of education and a prior history of depression were associated with AD, necessitating a comprehensive understanding and targeted interventions to achieve a global outcome [48,49].

Management

Management of NPS in dementia should prioritize non-pharmacologic interventions before considering drug therapy. Psychosocial interventions, such as environmental modifications (addressing overstimulation or under-stimulation), social interaction, music therapy, and caregiver training, have shown promise in improving behavior and mood in patients with dementia. A recent update to these techniques is the DICE approach, which involves describing the context, environment, and perspectives of patients and caregivers; assessing the degree of distress; investigating potential causes such as pain, fear, boredom, or environmental stimuli; creating an intervention plan; and evaluating any arising problems to determine whether to continue or modify the intervention [50,51].

The recommended plan of action includes establishing safety issues and NPS, implementing non-pharmacological strategies using the DICE approach, considering pharmacological approaches, always in combination with non-pharmacological interventions, reviewing the risks of medications, implementing any necessary therapies, and making informed decisions by discussing the risks and benefits for the individual [50-54].

These interventions can be effective in reducing symptoms, followed by medication as necessary. However, pharmacologic treatments, including atypical antipsychotics like risperidone and aripiprazole, have been associated with modest efficacy in treating symptoms such as psychosis, agitation, and aggression but carry significant risks of adverse events, such as seizures, stroke, and mortality. Regular evaluations of the risks and benefits during the course of treatment are essential. Regarding typical antipsychotics, a placebo-controlled trial of thioridazine found that patients receiving these medications showed improvement in agitation scores. Additionally, antidepressants like citalopram have been shown to reduce agitation but may worsen cognition and cause QT prolongation. Anti-dementia drugs, such as cholinesterase inhibitors (ChEIs) (e.g., donepezil, rivastigmine, and galantamine), have shown improvement in mild to moderate dementia and have effects on negative symptoms (e.g., apathy, irritability, and depression). Selective serotonin reuptake inhibitors (SSRIs) have been effective in treating agitation and anxiety but come with adverse effects, such as QT prolongation and hyponatremia [55-57].

Neurobiology of Alzheimer's disease

The neurobiology of AD involves multiple hypotheses, including the amyloid hypothesis, tau hypothesis, oxidative stress, mitochondrial hypothesis, inflammatory hypothesis, epigenetics hypothesis, cholinergic hypothesis, dendritic hypothesis, and the 5-HT6 receptor hypothesis [57-64]. These hypotheses propose mechanisms underlying the formation of amyloid plaques, neurofibrillary tangles (NFT), oxidative damage, inflammation, epigenetic modifications, Fyn activation, and the reduction in the production and transmission of acetylcholine, as well as dendritic disturbances, all of which contribute to the progression of AD.

The neurotransmitter systems implicated in AD, including cholinergic, serotonergic, and glutamatergic signaling, exhibit dysregulation in the disease process [65-67]. Cholinergic dysfunction, characterized by the degeneration of basal forebrain cholinergic neurons, is associated with cognitive decline in AD [65]. Alterations in serotonergic signaling, particularly involving 5-HT6 receptors, may contribute to the behavioral aspects of AD progression (66). Glutamatergic excitotoxicity, mediated by NMDA receptors, plays a role in neuronal loss and cognitive decline in AD [68].

Evidence supports dopaminergic neuronal degeneration in AD, particularly in the nigrostriatal pathway, marked by NFT, amyloid-beta (Aβ) plaques, neuropil threads, neuronal loss, and reduced dopamine content [69]. Dopaminergic receptors, including D1-5 receptors, play roles in learning, memory, and motivation [70-72]. The loss of D2 receptors in temporal and hippocampal regions suggests involvement in memory consolidation [73].

Beta-Adrenergic Receptors in Alzheimer's Disease

Beta2-adrenergic receptors play an important role in AD. Compared to the thalamus of control brains, the thalamus of brains with dementia had a lower total concentration of beta-adrenergic receptors. In comparison to control brains, brains with dementia have significantly lower concentrations of beta1-adrenergic receptors in the hippocampus and higher concentrations in the nucleus basalis of Meynert (NbM) and cerebellar hemisphere. Brains with dementia also show lower concentrations of beta2-adrenergic receptors in the thalamus, NbM, and cerebellar hemispheres but higher concentrations in the hippocampus and putamen [74]. The loss of noradrenergic locus coeruleus neurons in AD may exacerbate amyloid plaque formation and the severity of dementia [75]. Locus coeruleus projections facilitate Aβ uptake and clearance by microglia, linking noradrenergic signaling to AD pathology [76]. Soluble Aβ-induced alterations in locus coeruleus neurons suggest a link between noradrenergic signaling and inflammation in AD [77].

Pharmacological treatment and clinical outcomes in Alzheimer's disease

AD progressively impairs cognition and behavior, necessitating outcome measures that reflect its clinical hallmarks. Assessment scales evaluate domains such as quality of life, cognition, clinicians' and caregivers' impressions of change, functional abilities, and behavior [78]. Pharmacological treatments for BPSD have been reviewed recently, showing a major impact on various spectrums of AD [79]. Cognitive outcomes include measures such as the ADAS-Cog, Mini-Mental Status Examination (MMSE), Severe Impairment Battery (SIB), and Cambridge assessment of memory and cognition. Global measures comprise the Clinical Global Impression of Change (CGIC) and the Clinician Interview-Based Impression of Change (CIBIC), as well as the global deterioration scale. Functional measures include the Progressive Deterioration Scale (PDS), Alzheimer’s Disease Cooperative Study Activities of Daily Living inventory (ADCS-ADL), and Disability Assessment for Dementia (DAD) scale. Behavioral measures include the Neuropsychiatric Inventory and Cohen-Mansfield Agitation Inventory [79-81].

Vascular Prevention

When addressing vascular risk factors, the two main factors of concern in relation to cognitive outcomes are hypertension and dyslipidemia. Three randomized controlled trials have demonstrated that treatment of these factors, whether or not cerebrovascular disease is present, can positively impact cognitive outcomes. While some studies suggest benefits from antihypertensive agents, others, like the HYVET trial, show no reduction in cognitive impairment but do demonstrate significant impacts on cardiovascular and cerebrovascular outcomes. The CCCDTC (Canadian Consensus Conference on the Diagnosis and Treatment of Dementia) gave hypertension treatment a GRADE B, Level 1 recommendation as an option for preventing cognitive decline. Cholesterol-lowering agents in individuals at risk have not shown a significant impact on cognitive outcomes. However, treatment of vascular risk factors in individuals with AD correlates with slower cognitive decline, as per observational studies. For example, a longitudinal observational study in a tertiary clinic showed that treating vascular risk factors is associated with a slower decline in cognitive impairment in people with AD [79].

Cholinesterase Inhibitors

Neurochemical studies reveal cholinergic dysfunction in AD, leading to trials on ChEIs. Donepezil, rivastigmine, and galantamine are widely used. Donepezil, a piperidine derivative that inhibits acetylcholinesterase (AChE), shows cognitive improvement in AD, especially in terms of functional and behavioral outcomes, though it is associated with dose-dependent side effects such as nausea, vomiting, and muscle cramps. Rivastigmine, a carbamate derivative that inhibits both acetyl- and butyryl-cholinesterase, exhibits cognitive benefits at high doses. Pooled analyses have shown improvement in cognitive, global, and functional outcomes, but side effects are also dose-dependent. Nowadays, transdermal patches are preferred over oral formulations due to their association with three times fewer gastrointestinal cholinergic side effects. Galantamine, a tertiary alkaloid and reversible AChE inhibitor, also has an allosteric binding mechanism at nicotinic receptors, enhancing cholinergic transmission and improving cognitive function in mild to moderate AD and mixed dementia. It shows sustained cortical inhibition of AChE [79].

Comparative Studies of Cholinesterase Inhibitors

Comparison trials of ChEIs in AD yield conflicting results due to methodological limitations. Meta-analyses indicate differences in tolerability, favoring donepezil over rivastigmine in terms of adverse event frequency. However, rivastigmine as a transdermal patch is used in all stages of AD, while its oral form has reported more adverse events. Among ChEIs, agent selection should be based on adverse effects, ease of use, and other pharmacokinetic differences, as well as the stage of AD. Notably, ChEIs like rivastigmine, galantamine, and donepezil are approved by the FDA for mild to moderate AD treatment. Donepezil in its oral form and rivastigmine as a transdermal patch are used across the full spectrum of AD stages [80].

Memantine

Memantine, a non-competitive N-methyl-D-aspartate (NMDA) glutamate receptor antagonist, shows benefits in moderate to severe AD, with significant improvements in cognition, function, and global impression [80]. However, in a pairwise meta-analysis, memantine 20 mg daily alone or in combination with donepezil 10 mg was superior to placebo for assessing cognition in moderate to severe AD. ChEIs were less tolerated than placebo in mild to moderate AD. According to the SUCRA rankings, combination treatment for cognitive functions in moderate to severe AD is the best option, followed by donepezil 23 mg daily. While memantine is generally well-tolerated, it may increase the risk of agitation compared to placebo. The study revealed that in terms of tolerability in mild to moderate AD, the best regimen was memantine 20 mg daily followed by donepezil 5 mg. For moderate to severe AD, the study showed that memantine 20 mg daily had more significant potential adverse effects than placebo, and rivastigmine in oral form was also associated with high levels of adverse effects. As for the scales, CIBIC-plus and CGIC indicated that ChEIs like donepezil (5 mg, 23 mg, and 10 mg) showed significant efficacy compared to placebo, even rivastigmine 12 mg (10 cm² and 15 cm² patch) was superior to placebo. Rivastigmine received the highest treatment acceptability, followed by donepezil. ChEIs have shown better outcomes in mild to moderate patients, particularly in terms of cognition. In moderate to severe AD, combination therapy is preferred. ChEIs, like higher-dose rivastigmine transdermal patches, have shown significant improvement in function and clinical global impression, while memantine shows overall best acceptability [80,81]. The rivastigmine transdermal patch has better tolerance than the oral form and has been approved for use in all stages of AD. Cost-effectiveness evaluations of AD treatments, particularly ChEIs like donepezil and memantine, showed good results and better quality-adjusted life years (QALY) [80]. However, the degree of uncertainty varies from country to country. Latrepirdine, an antihistamine agent, showed promise in AD and Huntington's disease treatment, but it failed in phase 3 trials for both conditions after showing good efficacy in phase 2 trials. However, it was seen to work on multireceptor activity, calcium catabolic pathways, and mitochondrial function [81].

Advancements in Alzheimer's Disease Therapeutics

Research into AD therapeutics has focused primarily on two main classes: AChE inhibitors and NMDA receptor blockers. While atypical neuroleptics and serotonin-modulating antidepressants also show promise, their clinical effectiveness remains limited [82]. Recent efforts have shifted toward developing new strategies for AD treatment [83], such as 7-methoxy tacrine (7-MEOTA) derivatives targeting multiple pathways [84]. Antioxidants, including Vitamin E and Ginkgo biloba, have shown some cognitive benefits, with vitamin E suppressing tau-induced neurotoxicity. Caffeine has also exhibited anti-neuroinflammatory properties and reduced tau protein phosphorylation in the hippocampus [85,86]. Ubiquinone and omega-3 fatty acids have yielded mixed results, although omega-3 fatty acids are said to decrease the risk of AD [87,88]. Non-steroidal anti-inflammatory drugs (NSAIDs), like the R-enantiomer of flurbiprofen, have shown promise but face challenges in clinical trials [89,90]. Atypical antipsychotics and antidepressants are used to manage behavioral and psychological symptoms of AD, with citalopram showing promise but being associated with adverse effects [91-93]. HMG-CoA reductase inhibitors, or statins, have demonstrated potential in reducing AD progression through cholesterol-lowering effects and other mechanisms [94,95]. Hormone replacement therapy, particularly testosterone, has shown cognitive improvement in male AD patients [96]. 7-MEOTA derivatives represent a multi-targeted approach to AD treatment, addressing complex pathological pathways [97]. These novel therapeutic strategies hold promise but require further clinical validation for effective AD management.

Transcranial magnetic stimulation

History of Transcranial Magnetic Stimulation

The history of transcranial magnetic stimulation (TMS) traces back to ancient Roman times, when Scribonius Largus used electric torpedoes for medical purposes, marking the early use of electricity in medicine. In the 17th century, William Gilbert introduced the term "electricity," and in the 18th century, Krueger proposed using electricity for paralysis treatment. Significant advancements occurred when Luigi Galvani discovered bioelectricity in 1771, and Michael Faraday observed magnetic fields generated by electrical currents in 1831, laying the foundation for TMS. In 1896, French physician d’Arsonval reported the induction of phosphenes by magnetic fields, and in 1902, Beer claimed to treat depression using electromagnetic induction. The development of electroconvulsive therapy in 1937 marked a pivotal moment in brain stimulation. The first magnetic stimulation of human nerves occurred in 1965, and in 1985, Anthony Barker and colleagues built the first reliable TMS device. Further advancements led to the approval of rTMS by Health Canada in 2002 and the U.S. FDA in 2008 for treating major depressive disorder. Milestones include Dr. Alvaro Pascual‐Leone's work on speech arrest induction with rapid‐rate TMS in 1991, which paved the way for nuanced studies of cognitive function and neural interaction. The establishment of guidelines for TMS application in 1998 marked significant progress in brain stimulation research. The storied history of TMS shows how insights from various fields can lead to translational breakthroughs. Understanding the maturation and ongoing refinement of TMS is instructive in predicting how the field may evolve in the future [98,99].

Principles of Repetitive Transcranial Magnetic Stimulation

TMS is a non-invasive technique that stimulates brain tissue through high- and low-intensity magnetic fields, which modulate cortical excitability and neural plasticity. rTMS involves applying repeated TMS pulses to a specific brain region. rTMS can be categorized by frequency: high-frequency rTMS (>1 Hz) increases cortical excitability, while low-frequency rTMS (<1 Hz) decreases it [100]. TMS operates on Michael Faraday’s law of electromagnetic induction, which involves the passage of a high-intensity brief current through a copper wire, converting extracranial electrical energy into intracranial current [100].

Lenz's law, derived from the principle of energy conservation, states that a time-varying current induces a magnetic field, which in turn generates an electric field in nearby conducting mediums. The stimulating coil, through which electricity flows, creates a magnetic field that induces eddy currents in bodily tissues, responsible for therapeutic effects [100]. rTMS, as a brain stimulation technique, involves seating a patient with a large wire coil placed on the scalp. The magnetic field induces an electric field sufficient to depolarize superficial axons and activate cortical neural networks, causing cortical excitability. This tapping sensation is felt against the patient’s head, also known as mapping. During the procedure, the doctor determines the amount of magnetic energy by increasing the dose until a finger or hand twitch is observed. This procedure can be routinely practiced as an outpatient department (OPD) procedure, after which the patient can continue their daily activities [100]. The nature of the neural circuit, including parameters such as coil type, orientation, distance from the brain, and stimulation pattern, influences the extent of current density and stimulation effects [100].

Mechanism of Action of Repetitive Transcranial Magnetic Stimulation

rTMS can shift the transmembrane potential of neurons, leading to depolarization, sodium channel opening and subsequent action potential generation, changes in excitatory thresholds, and synaptic efficacy. rTMS-induced LTP is usually related to pulse frequency. For instance, 1 Hz rTMS for more than 10 minutes produces long-term depression (LTD) outcomes, while 5 Hz rTMS produces effects similar to LTP. Changes in cellular calcium levels and the strength of intracellular culture flow across the postsynaptic membrane could be related to LTP or LTD expression. Studies have demonstrated that rTMS electric signals cause astrocytes to respond to neuronal activation, playing a major role in neural circuit formation. Hence, rTMS regulates astrocytes in terms of nerve connections and in response to damage. Metaplasticity, a higher-order form of synaptic plasticity, is modulated by pre-stimulatory activity. Studies have shown that rTMS regulates the activation of NMDA receptors through G-coupled protein receptors and other cycles that increase levels of postsynaptic intracellular calcium [101,102].

High-frequency rTMS induces cortical excitation. When applied to the primary motor cortex, rTMS can provoke muscle movement and alter cardiac responses mediated by connections in the brain related to cardiac centers. It can also cause heart rate variability, suggesting autonomic nervous system modulation. It could cause cardio acceleration or inhibition, but it remains to be determined whether it can help predict cardiac ailments [103].

rTMS affects the hypothalamic-pituitary-adrenocortical system, neurotransmitter release, synaptic efficiency, signaling pathways, and gene transcription. It can normalize stress-induced hormone elevation and increase brain-derived neurotrophic factor (BDNF) levels, which have shown evidence of rescuing neuronal cell death caused by ischemic injuries, hypoglycemia, and others. Its neuroprotective, anti-inflammatory, and antidepressant nature aids in mood regulation [103]. Another animal study showed that rTMS promoted the upregulation of neural stem cells and increased BDNF and phosphorylated p-ERK 1/2 in the hippocampus [104].

Dopamine release increases in response to prefrontal cortex stimulation, impacting mood and motivation. Studies have shown that prefrontal rTMS can induce dopamine release in mesostriatal, mesolimbic, and striatal regions, supporting the hypothesis that rTMS affects dopamine levels and may help treat major depressive disorders [105].

Serotonin receptor upregulation and downregulation are associated with rTMS treatment outcomes in depression. Serotonin, an excitatory transmitter, is decreased in the hippocampus and prefrontal cortex of patients with depressive disorders. rTMS helps upregulate serotonergic transmission in major depressive disorders, whereas exposure to chronic rTMS causes 5HT1-A and 5HT1B autoreceptors, which usually control 5HT levels in the frontal cortex, to be reduced. Glutamate and GABA-mediated neurotransmission alterations contribute to the therapeutic effects of rTMS. These mechanisms collectively underscore the diverse physiological changes induced by rTMS, highlighting its potential in neuromodulation and therapeutic applications [106].

Special Considerations for Patients With Metal Implants

RTMS for individuals with metal implants can be challenging but is sometimes performed. Metal implants close to the stimulating coil, such as orthopedic implants, aneurysm clips, or residual metal fillings in the eye, may cause issues related to heat or displacement during stimulation. RTMS in patients with metallic elements within the brain is generally not advised, except for certain cases. For instance, metal implants without attached wires, such as aneurysm clips or metallic sutures, may be acceptable if they are not contraindicated for MRI (e.g., titanium). Therefore, a reliable questionnaire should be administered before stimulation. Valves used to treat hydrocephalus are considered safe, though experimental checks of valve placement before stimulation are recommended. Cardiac pacemakers, vagus nerve stimulators, or deep brain stimulators are not contraindications but should be placed more than 10 cm away from the stimulation site to avoid harm. In uncertain cases, therapy should be avoided [104]. Ideally, RTMS in patients with metal implants is not advisable. However, procedures such as ex vivo stimulation of an element similar to the one implanted, observing possible displacement or heating, can be conducted [107].

Effect of transcranial brain stimulation on Alzheimer's disease

Given the limited effectiveness of pharmaceutical treatments, there is growing interest in non-pharmacological approaches for AD. Recent non-invasive neurostimulation techniques, such as rTMS and transcranial direct current stimulation (tDCS), have emerged. rTMS enhances cortical and neural plasticity, while anodal tDCS has been shown to improve memory and cognitive functions. tDCS can significantly enhance working memory in older patients and alter resting-state functional connectivity in frontoparietal brain regions [9,72]. Both methods have demonstrated potential therapeutic benefits in cognitive neuroscience. The application of these techniques has expanded to various neurological and psychiatric conditions. Similarly, tDCS has shown promise in improving cognition in patients with neuropsychiatric disorders such as depression, Parkinson’s disease, and stroke [108].

Neuromodulatory Techniques

Neuromodulatory techniques like rTMS and tDCS offer non-invasive methods to modulate cortical excitability and induce lasting effects. rTMS delivers single TMS pulses in trains with consistent frequency and intensity, while tDCS applies low-intensity electrical currents via large saline-soaked sponge electrodes. Typically, low-frequency rTMS and cathodal tDCS aim to reduce cortical excitability, whereas high-frequency rTMS and anodal tDCS seek to enhance it. Although the exact mechanisms of rTMS and tDCS remain incompletely understood, both techniques impact synaptic plasticity, including LTP and LTD. Recent research has linked rTMS effects with synaptic plasticity and suggested that tDCS modulates synaptic strength within the cortex through intracortical neurons [105].

Repetitive Transcranial Magnetic Stimulation

A recent meta-analysis by Guse et al. (2010) [109] supports the positive impact of rTMS on various cognitive functions, including executive functions, learning, and memory. This study involved a 73-year-old patient with dementia and a mental disorder who was treated with rTMS despite poor drug response. The initial treatment with 1 Hz rTMS improved cognitive and behavioral symptoms and sleep duration. After a month, 10 Hz rTMS was tried, which normalized the sleep cycle and reduced cognitive decline, although seizures occurred, leading to a reduction in frequency to 0.8 Hz. This adjustment resulted in a significant positive response [110-112].

According to Cotelli et al. (2011), rTMS significantly improved language comprehension in AD patients [113]. Zhang et al. (2019) supported this finding, showing significant memory improvement with real TMS compared to sham TMS (real TMS-CT change: 3.87±0.82 vs. sham TMS-CT change: 0.29±1.07) [111]. Padala et al. (2020) used CGI-I and CGI-S indices to assess AD patients' mental status, revealing profound improvements with rTMS treatment [114]. In two crossover, sham-controlled, single-session studies, rTMS administered to the dorsolateral prefrontal cortex (DLPFC) during naming tasks showed significantly improved accuracy in action naming. The absence of rTMS effects on object naming in early-stage AD may be due to a “ceiling” effect, while moderate to severe AD patients showed improvements [112,113]. The bilateral facilitation effect observed in both mild and severe AD patients suggests compensatory mechanisms involving right hemispheric resources. Bentwich et al. (2011) [112] demonstrated promising results from combining rTMS with cognitive training (rTMS-COG), enhancing cognitive functions. The insights on the therapeutic effects of rTMS in AD largely stem from the work of Cotelli et al. (2008) [113], who conducted three consecutive studies assessing the impact of high-frequency rTMS on naming and language performance in AD subjects. These studies demonstrated improvements in naming and language tasks, particularly in mild AD patients, with implications for the specificity of rTMS effects on the language network.

Transcranial Direct Current Stimulation

In a pilot study by Ferrucci et al. (2008) [115], the hypothesis that anodal tDCS over the temporoparietal cortex could enhance cortical function and improve recognition memory in AD patients was tested. Ten patients with mild AD received anodal tDCS, cathodal tDCS, and sham tDCS over bilateral temporoparietal areas in separate sessions (15 minutes at 1.5 mA, with at least one-week intervals). Anodal tDCS significantly improved recognition memory, while cathodal tDCS decreased accuracy in word recognition tasks, and sham tDCS had no effect. Importantly, no adverse events were reported. In another study by Boggio et al. (2009) [116], patients with mild to moderately severe AD were exposed to anodal tDCS over the left DLPFC, anodal tDCS over the left temporal cortex (with the cathode electrode over the right supraorbital area for both sessions), or sham stimulation. Given that declarative memory is notably impaired in AD, the study aimed to assess the impact of anodal tDCS on recognition memory, working memory, and selective attention. Sessions were spaced 48 hours apart, and patients were tested during each stimulation session, starting 10 minutes after stimulation onset and lasting until the end (30 minutes at 2 mA). Stimulation over both prefrontal and temporal areas significantly improved visual recognition memory without affecting working memory. However, most prior studies lack robust evidence, and the notion that enhancing cortical plasticity could ameliorate the cognitive status of AD patients remains speculative. While studies have indicated cortical hyperexcitability in AD, therapeutic interventions targeting increased cortical excitability (such as anodal tDCS and high-frequency rTMS) may be based on assumptions and require further investigation. The relationship between cortical excitability and the brain's state during stimulation remains debatable, and the assertion that plasticity mechanisms are diminished in AD-affected brain regions, such as the DLPFC or temporoparietal regions, remains inconclusive. Therefore, a deeper understanding of cortical physiology and comprehensive pre- and post-therapeutic intervention assessments are warranted.

Side Effects of Repetitive Transcranial Magnetic Stimulation

There are multiple side effects of rTMS. Headache, the most common side effect, is usually mild and transient and occurs in up to 50% of patients. Scalp discomfort, such as tingling, burning, or itching at the stimulation site, is typically mild and transient. Lightheadedness or dizziness is uncommon but can occur, particularly with higher stimulation intensities. Facial muscle twitches are uncommon and usually brief and self-limiting. Hearing clicks or buzzing may occur during stimulation due to magnetic field interaction with the auditory system. Cognitive changes, including short-term memory problems or difficulty concentrating, have been reported in a small number of cases. Seizures are a rare but serious potential side effect, especially in individuals with a history of epilepsy or other seizure risk factors [117].

Limitations and future directions

One limitation of this review is the lack of a systematic methodology, which may introduce selection bias and result in incomplete coverage of relevant studies. Without quantitative synthesis, drawing conclusive insights into rTMS efficacy for AD treatment is challenging. Subjective interpretation and narrow inclusion criteria may also limit the review's comprehensiveness and generalizability. Future research should focus on optimizing TMS parameters, such as frequency, number of trains, train duration, inter-train interval, and overall pulses and sessions, specifically for AD effectiveness while minimizing adverse events. Long-term follow-up studies are needed to explore the duration of rTMS effects and maintain gains. Further refinements could involve individual MRI for neuronavigation to enhance precision in targeting specific brain areas. Incorporating EEG with rTMS may offer insights into electrophysiological activities during stimulation. These advancements could contribute to more personalized, targeted interventions for patients with AD. Ultimately, well-designed randomized controlled trials will be essential to establish clinical guidelines and regulatory approval for rTMS in Alzheimer's care.

## Conclusions

This research suggests that rTMS could serve as an adjunctive treatment alongside cognitive enhancers for AD and dementia-related pathology, highlighting its potential therapeutic role. AD is a global concern, with a very high degree of morbidity across the world. rTMS, as a newly evolving technique, promises functional improvement in AD. Preclinical models can be further explored to build a base for human clinical trials. The physiology of rTMS interactions can be studied in models and animal studies. Future research should focus on conducting multicentric trials across the globe, involving a diverse cohort of patients in order to generate rigorous evidence. 
